# Antibiotic Resistance Patterns of *Escherichia coli *Isolated from Children in Shahid Sadoughi Hospital of Yazd

**Published:** 2013-04-22

**Authors:** J Ayatollahi, S H Shahcheraghi, R Akhondi, SS Soluti

**Affiliations:** 1 Infectious and Tropical Diseases Research Center, Shahid Sadoughi University of Medical Sciences and Health Services and Health Services, Yazd, Iran.; 2 Department of Modern Sciences & Technologies, Faculty of Medicine, Mashhad University of Medical Sciences, Mashhad, Iran.

**Keywords:** Escherichia coli, Drug Resistance, Microbial, Child

## Abstract

**Background:**

Growth of drug resistance is related to number of microbial characteristics, selective pressure by antibiotic use and social and technical vicissitudes that enhance the transmission of antibiotic resistant organisms. The aim of this study was to investigate antimicrobial-resistance of *Escherichia coli* isolated from children in Shahid Sadoughi hospital of Yazd.

**Materials and Methods:**

In this cross-sectional study, antimicrobial susceptibility to cefixime , cefotaxime , ceftazidime , ceftriaxone, ciprofloxacin, gentamicin, imipenem, cotrimoxazole and nalidixic acid was determined for 148 E. coli isolates obtained from patients less than 18 years of age (hospitalized and outpatient) in Shahid Sadoughi hospital of Yazd.

**Results:**

Ciprofloxacin was the most active antibacterial agent (78% susceptible), followed by gentamicin. High rates of resistance were observed for cefixime (57.9%) and cotrimoxazole. The results for ceftriaxone, cefotaxime, ceftazidime, ciprofloxacin, gentamicin, imipenem, cotrimoxazole and nalidixic acid were insignificant with P-value= 0.302, P-value= 0.550, P-value= 0.334, P-value= 0.084, P-value= 0.948, P-value= 0.686, P-value= 0.120 and P-value= 0.162, respectively. The results were significant for cefixime with P-value= 0.013.

**Conclusion:**

The investigation of antimicrobial susceptibility is essential, and will help to identify *E. coli* resistance to antimicrobial agents. It also helps to limit *E. coli* spread.

## Introduction

Antibiotics have played an important role in reducing sickness and mortality associated with infectious and tropical diseases in humans and animals. However, optional enforcement applied by the use of these agents the primary driving power behind the emergence and extension of antibiotic-resistance properties among all bacteria (1). Antimicrobial agents have primarily been used to cure infectious diseases caused by bacteria. Use of antibiotics is an important risk factor for extension of resistance to these agents (2). The number of bacteria that are resistant to antibiotics in the perimeter increase with the application of antibiotics (3-5). Antimicrobial agents- resistance spreads between various strains of bacteria in different environments (6-9). Propagation of resistant strains such as *Escherichia coli,* *Campylobacter *spp. and *Enterococcus *spp. is associated with keeping products such as poultry in contaminated sites (10-13). Pathogenic strains of *E. coli *cause infections including urinary tract infection, gastroenteritis, meningitis, septicemia and peritonitis (14, 15). *Escherichia coli* is one of the most important causes of morbidity and mortality throughout the world particularly in developing countries (16, 17). Remedial answers vary pertaining to the type of infection (18). Resistant *E.coli *to antibiotics is associated with decreases in clinical remedy levels and higher danger of regression (19-21). Ruzauskas et al investigated the prevalence and the antimicrobial resistance of *E. coli* isolates. The most frequent resistance was related to streptomycin, followed by ampicillin and nalidixic acid (22). Another study was performed by Akond et al to investigate antibiotic resistance of *E. coli* in Bangladesh. Fifty identified strains were subjected to examine their susceptibility to 13 antimicrobial agents. In Akond study, none of the strains showed resistance to gentamicin (23). A study was conducted to evaluate antibiotic sensitivity of *E. coli* strains isolated from several types of infected wounds. The results revealed a high sensitivity to amikacin and imipenem (24). Antimicrobial susceptibility of *Escherichia coli *and other coliforms isolated from asymptomatic male and female students of Niger Delta University in Bayelsa State, Nigeria has been investigated.The highest rate of sensitivity was to gentamicin (25). The aim of this study was to investigate antimicrobial-resistance of *Escherichia coli* isolated from patients less than 18 years of age in Shahid Sadoughi hospital of Yazd.

## Materials and Methods

This cross-sectional study was performed on 148 *E. coli* obtained from patients less than 18 years of age with positive cultures (hospitalised and outpatient) in the Laboratory of Shahid Sadoughi hospital of Yazd, from January 2010 to December 2011. The samples to obtain bacterial strain were urine, blood, and discharge and blood cultures in patients undergoing intra-arterial angiography (129 urine, 7 blood, 8 discharge samples and 4 angio sample). Examined antibiotic types in antibiogram test were including: 

cefixime , cefotaxime , ceftazidime , ceftriaxone, ciprofloxacin, gentamicin, imipenem, cotrimoxazole and nalidixic acid that were evaluated for antimicrobial-resistance in *E. coli*. In disk diffusion assay which was used in this study, within 15 min after applying the antibiotic discs (Oxoid, Australia Company) the plates were inverted and incubated at 37 °C. After 24h of incubation, the plates were examined, and the diameters of the zones of complete inhibition to the nearest whole millimeter were measured. The zone diameter for individual antimicrobial agents was then translated into susceptible, intermediate and resistant categories. The information was first collected from Shahid Sadoughi hospital laboratory.

**Statistical Analysis**

The collected data was analyzed with the software SPSS, using chi-square test.We also obtained the sample size by cochran sampling formula**.**

## Results

The present study included antimicrobial susceptibility data for *E. coli *isolates obtained from patients hospitalized for 48 hrs or more with positive cultures and outpatient in Shahid Sadoughi hospital of Yazd. The ages of patients in our study ranged from 1 year to 17 years (Table I). Susceptible(S), intermediate (I) and resistant (R) percentages of the isolates to the antimicrobial agents were shown in Table II. The highest rates of resistance were for cefixime (57.9%) and cotrimoxazole (54.7%). Low levels of resistance were against ciprofloxacin (78%) and gentamicin (66.9%). The results for ceftriaxone, cefotaxime, ceftazidime, ciprofloxacin, gentamicin, imipenem, cotrimoxazole and nalidixic acid were insignificant with P-value=0.302, P-value=0.550, P-value=0.334, P-value=0.084, P-value=0.948, P-value=0.686, P-value=0.120 and P-value=0.162, respectively. The results were significant for cefixime with P-value=0.013.

**Table I:** The distribution of patients' ages. The table shows the number and percentage of patients in the age groups 1-17 years old.

**Patient Age Categories (** **year) **	**No. of Cases ** **n** ** (%)**
1-6	96 (64.84)
5-10	36 (24.32)
10-15	5 (3.37)
15-17	11 (7.43)

**Table II:** Antimicrobial susceptibility of E.coli isolates. This table shows the sensitivity against cefixime (37.2%), cotrimoxazole (40.1%), ciprofloxacin (78%) and gentamicin (66.9%).

**Antimicrobial agent**	**Resistant, n (%)**	**Susceptible, n ** **(%)**	**Intermediate, n (%)**	**Total , n(%)**
**Cefixime**	70(57.9)	45 (37.2)	6 (5)	121(100)
**Cefotaxime**	41 (36.6)	67 (59.8)	4 (3.6)	112(100)
**Ceftazidime **	44 (44.4)	47 (47.5)	8 (8.1)	99 (100)
**Ceftriaxone**	57 (41.6)	72 (52.6)	8 (5.8)	137(100)
**Ciprofloxacin**	20 (18.3)	85 (78)	4 (3.7)	109 (100)
**Gentamicin**	31 (22.8)	91 (66.9)	14 (10.3)	136 (100)
**Imipenem**	12 (14.1)	68 (80)	5 (5.9)	85 (100)
**Cotrimoxazole**	75 (54.7)	55 (40.1)	7(5.1)	137 (100)
**Nalidixic acid**	52 (59.8)	31 (35.6)	4 (4.6)	87 (100)

## Discussion

Infectious diseases by resistant bacteria have been an extraordinary fondness in all medical and therapeutic centers. The development of antibiotic-resistant bacterial strains is an appearing worldwide danger that increasingly menaces the successful remedy of diseases related to these strains. *E.coli* is one of antibiotic-resistant gram-negative bacteria in hospitals. This bacterium causes infections including urinary tract infection; gastroenteritis, septicemia and meningitis. In Lithuania, the most frequent resistance of *E.coli* was for streptomycin (100 %) (22). Two hundred and forty samples of new raw chicken liver were obtained from national fowl producers in different badger marketing sites and tested for the presence of *E. coli*. One hundred *E. coli *strains were separated and tested for susceptibility against antibiotics. The study was performed to estimate the antimicrobial resistance of *E. coli *related to raw chicken liver in Lithuania. This study and our study approved resistance against nalidixic acid (59.8%) (22). In another study that was performed in the capital city of Bangladesh,the aim was investigation of antibiotic resistance of *E. coli* obtained from fowl sources of different markets. None of the strains showed resistance to norfloxacin and gentamicin.86%, 80%, 60%, 36%, 30%, and 26% of the strains were sensitive to norfloxacin, gentamicin and chloramphenicol, neomycin, tetracycline, streptomycin and ampicillin, respectively (23). Our findings also showed sensitivity against gentamicin (66.9%). In another study in Oradea, Romania, the sensitivity of antibiotics to *E. coli* strains was evaluated. *E. coli *variants were separated from several types of infected wounds in patients who were hospitalized in the emergency room of hospital.The outcomes showed the highest sensitivity (75%) to amikacin, between 35-50% to IV-th generation cephalosporins and 52.3% to imipenem. It has been shown a lower rate of sensitivity to gentamicin (38.6%) (24). All *E. coli *variants separated from surgical wounds were sensitive to amikacin, gentamicin, cefoperazone, ceftriaxone, imipenem and ciprofloxacin (24). In comparison with this study we have also demonstrated a high sensitivity against ciprofloxacin (78%), gentamicin (66.9%). In Niger Delta University in Bayelsa State, Nigeria, *E. coli *and other coliforms from midstream clean-catch urine samples of students were separated and tested for their susceptibility to commonly used antimicrobial agents. Resistances against several antibiotics were considered significantly in both *E*. *coli *(83.9%) and the unclassified coliforms (100%). In this study, the highest susceptibility was against gentamicin (64.5% for *E*. *coli *and 33.3% for unclassified coliforms) (25). Comparison of this study with present study showed that in the present study a high sensitivity against gentamicin (66.9%) was also demonstrated.Therefore, the results of this study represent high level resistant of *E. coli *isolates against cefixime and cotrimoxazole. It is because of inappropriate and incorrect administration of antimicrobial agents. This problem remarks significance of performing antimicrobial susceptibility testing before empiric antibiotic therapy. To overcome this problem; use of unnecessary antibiotics therapy should be limited.

## Conclusion

The investigation of antimicrobial susceptibility is essential because it will help to identify *E. coli* resistance to antimicrobial agents.In this study,the highest rates of resistance were to cefixime (57.9%), cotrimoxazole (54.7%). Low levels of resistance were to ciprofloxacin (18.3%), gentamicin (22.8%).
